# Multiplex Polymerase Chain Reaction for Detection of Gastrointestinal Pathogens in Migrant Workers in Qatar

**DOI:** 10.4269/ajtmh.16-0464

**Published:** 2016-12-07

**Authors:** John M. Humphrey, Sanjay Ranbhise, Emad Ibrahim, Hamad E. Al-Romaihi, Elmoubasher Farag, Laith J. Abu-Raddad, Marshall J. Glesby

**Affiliations:** 1 Division of Infectious Diseases, Department of Medicine, Weill Cornell Medicine, New York, New York; 2 Infectious Disease Epidemiology Group, Weill Cornell Medicine, Doha, Qatar; 3 Qatar Red Crescent Worker's Health Center, Doha, Qatar; 4 Hamad Medical Corporation, Doha, Qatar; 5 Communicable Diseases Department, Ministry of Public Health, Doha, Qatar; 6 Department of Healthcare Policy and Research, Weill Cornell Medicine, Cornell University, New York, New York

## Abstract

The causes of infectious diarrhea among the migrant worker population in Qatar are not well understood. We conducted a prospective observational study to understand the demographic and clinical characteristics and infectious causes of diarrhea among migrant workers in Doha, Qatar. A total of 126 male workers presenting to the Qatar Red Crescent Worker's Health Center outpatient clinic or emergency department were studied over a 5-month period in 2015–2016. Epidemiologic surveys were administered to all subjects and the prevalence of 22 different stool pathogens was determined using multiplex polymerase chain reaction (PCR) (FilmArray^®^ Gastrointestinal PCR). A target pathogen was identified in 62.7% of subjects. Enteropathogenic *Escherichia coli* was the most prevalent pathogen and was detected in 24.6% of subjects, followed by *Salmonella* (22.2%), enteroaggregative *E. coli* (15.1%), *Giardia lamblia* (9.5%), and enterotoxigenic *E. coli* (8.7%). Multiple pathogens were identified in 49.3% of positive stool samples. In a multivariable analysis, the presence of a heart rate ≥ 90 (adjusted odds ratio [OR] = 3.7, 95% confidence interval [CI] = 1.4–10.0) and > 5 fecal leukocytes/high-power field (adjusted OR = 2.8, 95% CI = 1.2–7.0) were significant predictors of detecting an acute inflammatory pathogen by PCR. Use of multiplex PCR enabled the detection of gastrointestinal pathogens in a high proportion of cases, illustrating the utility of this diagnostic tool in epidemiologic studies of infectious diarrhea.

## Introduction

Infectious diarrhea is an important cause of morbidity and mortality worldwide, resulting in considerable economic and public health burdens in both developed and developing countries.^1^–^4^ In Qatar and the greater Gulf region, few studies have been undertaken to characterize the epidemiology of infectious diarrhea.^5^,^6^ This is particularly the case among Qatar's migrant worker population, over half a million of whom are young and middle-aged men from the Indian subcontinent and other Asian countries who have come to work in Qatar's surging construction sector.^7^ Many of these individuals live in populous labor camps with dormitory-style housing, which pose risk for transmission of communicable diseases, including gastrointestinal pathogens. Indeed, data from Qatar's Ministry of Public Health suggest that a large proportion of the country's annual foodborne disease outbreaks occur in this community. From February 2013 to December 2014, for example, 17 suspected foodborne disease outbreaks were reported in Qatar and 15 of them occurred in the migrant worker community. Also, nearly 70% of 2,612 reported cases of foodborne illness from 2010 to 2014 occurred among the 20- to 39-year-old male migrant worker demographic (Qatar Ministry of Public Health, unpublished data). Concerns have been raised over the possibility of underdetection of foodborne disease outbreaks in the migrant worker community and over the possibility of imported gastrointestinal pathogens. In addition, the unprecedented mass gathering anticipated during 2022 World Cup further motivates the need to understand the current epidemiology of diarrheal diseases in the country.

Recently, multiplexed molecular diagnostics have become commercially available for the diagnosis of infectious diarrhea. These assays offer high sensitivity and specificity for a range of pathogens, providing a novel opportunity to narrow the diagnostic gap concerning the causes of infectious diarrhea.^1^,^8^ One such technology, the FilmArray^®^ Gastrointestinal polymerase chain reaction (PCR) is capable of detecting 22 different pathogens with a sensitivity ranging from 94.5% to 100% and specificity from 97.1% to 100% depending on the target.^9^ Such platforms are well suited for settings such as the migrant worker community in Qatar, for which broad diagnostic capacity near the point of care is currently needed to facilitate actionable data concerning the causes of potential foodborne disease outbreaks.^9^,^10^

Given the need for further data concerning the epidemiology of infectious diarrhea in the migrant worker population in Qatar, we conducted a prospective observational study aimed at describing the clinical features, epidemiologic characteristics, and etiologies of infectious diarrhea in this population using the FilmArray^®^ Gastrointestinal PCR.

## Materials and Methods

### Study site and ethical approval.

This prospective, community-based study was conducted at the Qatar Red Crescent (QRC) Workers Health Center, located in the New Industrial Area of Doha, Qatar. This facility provides outpatient and emergency department (ED) care exclusively to male migrant laborers, attending to 800–1,000 patients per day. This study was approved by the Institutional Review Board at Weill Cornell Medical College in Qatar (IRB no. 15-00051). All study participants provided written, informed consent.

### Enrollmentproceduresandinclusion/exclusioncriteria.

From August to September 2015 and January to May 2016, any individual coming to the clinic or ED with suspected infectious diarrhea was eligible to participate in the study. To be eligible for enrollment, subjects had to be ≥ 18 years of age and able to understand English, Arabic, Hindi, Malayalam, or Tagalog. These languages represented the majority of the clinic population and were based on the language capacity of the study personnel. For the study, presumptive infectious diarrhea was defined as three or more loose stools in a 24-hour period or two loose stools in a 24-hour period accompanied by other gastrointestinal symptoms such as nausea, vomiting, abdominal cramps, tenesmus, bloody stools, or fever that was defined as an oral temperature ≥ 38°C.^6^,^11^ Subjects were excluded from the study if they were diagnosed with a noninfectious cause of diarrhea, unable to provide a stool sample or complete the survey, or if the sample provided was formed stool (i.e., did not conform to the shape of the collection container). Of note, subjects were asked to submit a stool sample for PCR testing regardless of whether stool culture or microscopy was ordered by the subject's physician. We aimed to enroll 200 subjects to detect a *Salmonella* prevalence of 5% with a 95% confidence interval [CI] of 2–8%.^5^,^9^,^12^

### Demographic/clinical data collection and microbiological analysis.

A member of the study team administered a survey to enrolled subjects that assessed their demographic and clinical characteristics as well as a variety of risk factors for infectious diarrhea. The survey was an adaptation of the Minnesota Questionnaire, a standard foodborne disease outbreak case questionnaire, and translated into the aforementioned study languages using a certified translation service (Language Scientific, Medford, MA).^13^ Additional clinical and laboratory data (e.g., triage vital signs, medications prescribed, and pertinent laboratory results) were recorded retrospectively by accessing the patients' medical records. These data were entered into a Research Electronic Data Capture database.^14^ Stool samples were collected from study participants at the QRC clinic and immediately preserved in Cary-Blair enteric transport medium. The samples were then transported to Hamad General Hospital Microbiology Laboratory in Doha, Qatar, within 1–2 days, where they were tested with a commercially available multiplex PCR system, the FilmArray^®^ Gastrointestinal Panel (Biofire Diagnostics, Salt Lake City, UT). This assay consists of a self-contained, nested reverse transcription polymerase chain reaction reaction with melt analysis of the PCR product for analyte detection and automated results analysis. Each pouch also contains PCR and internal nucleic acid extraction controls.^15^ The multiplex PCR was validated according to the simple protocol in the manufacturer instructions before beginning the study.^16^ During the study, synthetic RNA quality controls were run for every 20 samples tested (Maine Molecular Quality Controls, Scarborough, ME). Because of the time required to transport the specimens to the facility where they were tested, PCR results were typically reported to the subject's physician within 2–4 days. Hence, treatment recommendations were made empirically by the subject's physicians at the time of the visit. Complete blood counts, stool microscopy, and stool culture were not performed as part of the study, but their results were recorded whenever these tests were ordered by the subject's physician. Stool microscopy and complete blood counts were performed at the QRC clinic, the former by a trained laboratorian immediately following sample collection. For stool culture, samples were transported to Hamad General Hospital Microbiology Laboratory and cultured for *Salmonella/Shigella* on Hektoen enteric agar, sorbitol MacConkey agar, and selenite broth.

### Statistical analyses.

Associations between various demographic and clinical variables and the detection of any pathogen by PCR were summarized with odds ratios (ORs) and 95% CIs. Each variable was treated dichotomously such that the reference group for each characteristic was all subjects who did not report that characteristic. Backward stepwise regression was used to construct multivariable models to identify factors predictive of a positive PCR result for any pathogen or one of the acute inflammatory pathogens (i.e., *Campylobacter*, *Salmonella*, *Shigella*, enteroinvasive *Escherichia coli* (EIEC), or Shiga-like toxin-producing *E. coli*).^17^ The following factors were included in the model: nationality, meal location (prepared at home versus cafeteria, catering company, or restaurant), reported symptoms, temperature ≥ 38.5°C, heart rate ≥ 90 beats per minute, ≥ 6 stools in a 24-hour period, presence of > 5 fecal leukocytes/high-power field,^18^ > 25 leukocytes/high-power field, fecal red blood cells or fecal mucous, and whether subjects received treatment in the ED. These factors were selected for conceptual reasons with a probability of removal from the model (P_r_) set at 0.1. Data were analyzed in STATA 14.1 (StataCorp., College Station, TX).

## Results

### Enrollment.

A total of 133 subjects were enrolled into the observational study. After enrollment, seven subjects were excluded because they did not submit a stool sample (*N* = 1), submitted formed stool (*N* = 4), or submitted an insufficient quantity of stool for PCR testing (*N* = 2). Thus, 126 subjects who completed the survey and submitted unformed stool were included in the analysis.

### Demographic characteristics.

Table 1 summarizes the demographic characteristics of the study subjects. All subjects were male and the median age was 33 years. All subjects were migrants, most were from the Indian subcontinent, and 6.3% reported having returned to Qatar from their home country within 21 days of clinical presentation for diarrhea. Nearly all subjects were from construction-related fields and were living in dormitories within worker camps, each individual sharing a room and bathroom with a median of five other individuals.

### Clinical characteristics.

Table 2 summarizes the clinical characteristics of study participants. Overall, 23.0% of subjects were evaluated and treated in the ED. Approximately 80% of individuals rated their baseline health as excellent or very good. Nine (7.2%) individuals reported a medical comorbidity, most frequently diabetes, and six (4.8%) reported a remote history of intra-abdominal surgery (appendectomy in all cases). No subjects were taking acid- or immunosuppressive medications at the time of presentation, and one individual reported taking an antibiotic for any reason within 3 months before developing diarrhea. The median duration of symptoms before clinical presentation was 2 days, and 54.8% of subjects reported a maximum of ≥ 6 stools in a 24-hour period, a cutoff suggested as an indicator of severe diarrhea.^19^ A total of 26.2% of evaluable subjects had a heart rate ≥ 90 beats per minute and 8.4% had a temperature ≥ 38.5°C.

### Laboratory and stool PCR results.

Table 3 summarizes the laboratory data and multiplex PCR results. Complete blood counts were performed in 31 subjects, of whom seven (22.6%) had white blood cell counts ≥ 15,000/mm^3^. Stool microscopy was performed in all but one study subject. One or more fecal leukocytes was detected in 90.4% of all stool samples and more than five leukocytes in 41.6% of samples. *Entamoeba* sp. and *Giardia lamblia* were identified by microscopy in 13.6% and 7.2% of samples, respectively. Stool culture was ordered for five subjects and was negative in all cases.

Overall, one or more pathogens was identified in 62.7% of cases. Enteropathogenic *E. coli* (EPEC) was the most prevalent pathogen and was identified in 24.6% of cases, followed by *Salmonella* in 22.2% of cases. Other diarrheagenic *E. coli* pathotypes were also prevalent, including enteroaggregative *E. coli* (EAEC, 15.1%), and enterotoxigenic *E. coli* (ETEC, 8.7%), and *Shigella*/EIEC (8.7%). Norovirus was identified in 4.0% and *Clostridium difficile* was not identified in any of the samples. *Giardia lamblia* was identified by PCR in 9.5% (*N* = 12) of cases, though 33% (4/12) of PCR-positive cases were positive by microscopy and an additional five cases reported by microscopy were negative by PCR. *Entamoeba* sp. was reported by microscopy in 17 cases, all of which were negative for *Entamoeba histolytica* by PCR. Among five subjects for whom stool culture for *Salmonella/Shigella* was ordered, PCR was negative in two cases, positive for *Salmonella* in one case, positive for *Campylobacter* sp. in another, and positive for EPEC, EAEC, ETEC, and EIEC in a third case. Throughout the study, each organism targeted by the PCR panel was successfully identified by synthetic RNA quality control panels without any misdetections (i.e., 10/10 successful positive and negative control panels). Four study samples required repeat PCR runs on account of initial invalid results due to software error.

Figure 1

**Figure 1.**
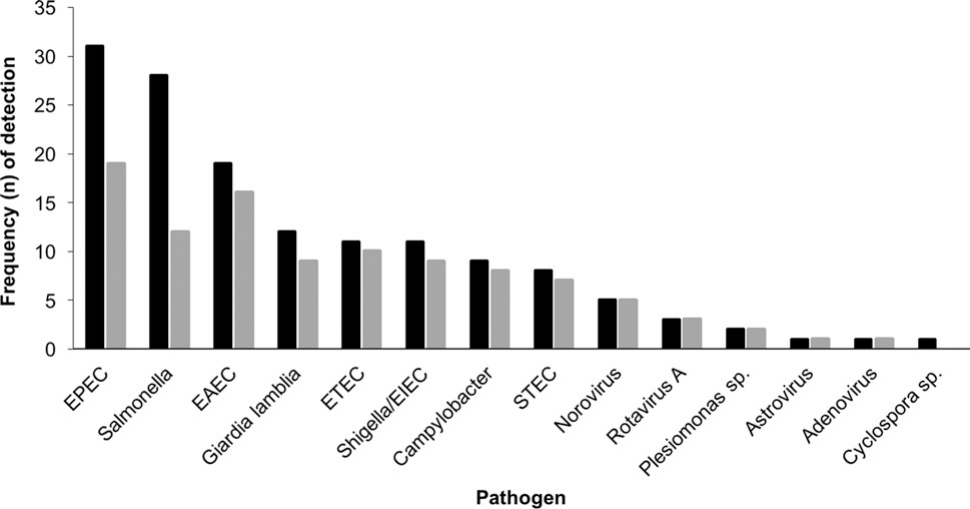
Frequency of detection and co-detection of potential pathogens among study subjects. Legend: Black bars represent total frequency of detection for each pathogen and grey bars represent frequency of co-detection in which one or more other pathogens were detected in the same stool sample. EAEC = enteroaggregative *E. coli*; EIEC = enteroinvasive *E. coli*; EPEC = enteropathogenic *E. coli*; ETEC = enterotoxigenic *E. coli*; STEC = Shiga-like toxin-producing *E. coli.*

depicts the frequency of pathogens detected by multiplex PCR along with the frequency of co-detection. Two or more potential pathogens were detected in 49.3% of positive samples (Table 3). Co-pathogens were detected for the majority of all pathogens with the exception of *Salmonella*, for which co-detection of multiple pathogens was identified in 43% of *Salmonella*-containing samples. The presence of abdominal cramps and a heart rate ≥ 90 beats per minute were the only statistically significant predictors of a positive PCR result for any pathogen in univariate analyses (Table 4). These covariates remained significant when adjusted for the aforementioned covariates in a stepwise multivariable analysis (heart rate ≥ 90 beats per minute, adjusted OR = 2.9, 95% CI = 1.0–8.5); abdominal cramps (adjusted OR = 3.3, 95% CI = 1.1–9.7). In a similar subanalysis to determine predictors of a positive PCR result for one of the acute inflammatory pathogens, only the presence of a heart rate ≥ 90 (adjusted OR = 3.7, 95% CI = 1.4–10.0) and > 5 fecal leukocytes/high-power field (adjusted OR = 2.8, 95% CI = 1.2–7.0) were significant covariates.

### Treatment.

A total of 67.5% of subjects received an empiric antibiotic, most commonly metronidazole (49.2%), ciprofloxacin (35.7%), or both in combination (18%) (Table 3). Metronidazole was prescribed in 46% (53/114) of cases where PCR was negative for *Giardia* (there were no detections of *C. difficile* or *E. histolytica*) and in 75% (9/12) of cases in which the PCR detected *Giardia*. Moreover, empiric ciprofloxacin or trimethoprim–sulfamethoxazole was prescribed in 43% (22/51) of cases in which no potentially susceptible bacterial pathogens targeted by the FilmArray^®^ PCR (e.g., *Salmonella*, *E. coli*, *Campylobacter*, among others) were detected.

## Discussion

Our study offers a detailed picture of the epidemiology of infectious diarrhea in the migrant worker population in Qatar. We found that *Salmonella* accounts for a high proportion of diarrhea cases, along with the diarrheagenic *E. coli* pathotypes EPEC, EAEC, and ETEC. Most of these infections would not have been ascertained under the present clinical and laboratory practices at the study site, in which stool culture is rarely ordered by physicians and testing for *E. coli* pathotypes, with the exception of *E. coli* O157, is not performed. In addition, the high overall pathogen detection rate of 62.7% in our study demonstrates the utility of multiplex PCR in epidemiologic surveillance studies and its potential to influence antibiotic prescribing practices for diarrheal diseases.

In many settings, the etiologies of diarrheal diseases remain poorly characterized due to limited surveillance and the low detection rates of traditional pathogen identification methods.^1^,^2^,^20^ In our study using multiplex PCR, 14 different pathogens were identified with an overall detection rate of 62.7%. This detection rate exceeds traditional laboratory detection methods^2^ and compares favorably to prior studies utilizing the FilmArray^®^ PCR in which detection rates ranged from 33% to 48%.^9^,^12^,^21^–^23^ Thus, the breadth of pathogens surveyed and ease of use of this PCR platform make it well suited for epidemiologic studies, particularly in settings such as ours in which the traditional suite of culture media types, immunoassays, and uniplex PCR protocols used to survey a similarly broad range of pathogens would be difficult to implement due to limited human and laboratory resources.

Another notable finding in our study was the 22.2% prevalence of *Salmonella* encountered in the study cohort. This prevalence exceeds those previously reported from cohorts of similar ages in Qatar^5^,^6^ and elsewhere in the United States and Europe, in which *Salmonella* prevalence ranged from 2% to 10% among both PCR- and culture-based studies.^9^,^12^,^20^,^24^,^25^ Nontyphoidal *Salmonella* is recognized as a major bacterial cause of infectious diarrhea and the most common bacterial cause of foodborne outbreaks, but its incidence has declined over recent years in Europe and the United States.^26^,^27^ In our study, whether the *Salmonella* cases were sporadic or outbreak related could not be determined given the absence of culture isolates from which to perform subtyping. However, the temporal pattern and variable demographic characteristics of observed *Salmonella* cases suggested that these infections were sporadic. Nevertheless, this finding warrants further research to clarify the epidemiology and risk factors for *Salmonella* infections in the migrant worker population and to evaluate the need for targeted prevention measures.

Diarrheagenic *E. coli* pathotypes were also commonly detected in our study population. This finding is consistent with prior surveillance studies utilizing multiplex PCR.^9^,^12^,^28^ Unlike studies in other settings, however, ours detected a relatively low frequency of norovirus and no cases of *C. difficile*. Our study definition may have influenced the former finding, as not including vomiting as a standalone inclusion criterion may have reduced the number of norovirus cases we were able to enroll. The absence of *C. difficile* is not unexpected given the study population of predominantly young, healthy adults without prior antibiotic exposure or contact with health-care environments where *C. difficile* is prevalent.

In our study, two or more potential pathogens were identified in 49.3% of positive samples and co-detection of multiple pathogens was present in the majority of cases for each pathogen except for *Salmonella* (see Figure 1). The high frequency of co-detection in our study, particularly among the diarrheagenic *E. coli* pathotypes, is similar to prior studies using the FilmArray^®^ PCR in which co-detections were reported in 21–48% of positive samples.^9^,^12^,^21^–^23^ Although co-detections in diarrheal diseases will likely be more commonly recognized with the increasing use of multiplexed molecular assays, whether such detections represent true coinfections of viable organisms or colonizers cannot be determined. Moreover, in the case of pathogenic *E. coli*, horizontal transfer of target genes located on plasmids or pathogenicity islands may account for the detection of multiple strains in a single sample.^29^

Refining diagnostic algorithms for stool testing remains important given the low positivity rate of traditional methods and the high cost of PCR testing.^2^,^30^ In separate multivariable analyses to determine predictors of a positive PCR result for any pathogen or one of the acute inflammatory pathogens, the presence of a heart rate ≥ 90 beats per minute was a significant predictor in both analyses, likely indicating the presence of dehydration and/or inflammation. The precision of these estimates was low and our small sample size limited power to detect significant effects in these analyses. The presence of fecal leukocytes on stool microscopy is also commonly used to suggest the presence of an invasive gastrointestinal pathogen, increasing the likelihood of a positive stool culture and influencing antibiotic decision-making. In our study, a threshold of > 5 leukocytes/high-power field was a significant predictor of detecting an inflammatory pathogen by PCR. Although this finding is supported by some studies,^18^ the presence of fecal leukocytes is known to lack sensitivity since many forms of colitis occur focally, which may be further confounded by the detection of low microbe burdens by highly sensitive PCR-based assays.^19^

Finally, the high proportion of subjects who received empiric antibiotics raises the potential for broadly multiplex PCR to influence antibiotic use for infectious diarrhea as well as the need for antimicrobial stewardship in community-based health-care settings. Empiric antibiotic treatment of infectious diarrhea should usually be reserved for febrile dysentery or suspected systemic infection, severe travelers' diarrhea, or health care-associated diarrhea suspected to be caused by *C. difficile*.^2^,^19^,^31^ In our population of predominantly healthy young men without immune-compromising conditions, 67% were empirically prescribed an antibiotic, most commonly ciprofloxacin and/or metronidazole. Receipt of either of these medications was not associated with having a PCR result positive for any of the organisms for which these antibiotics have activity. Quinolone resistance has been documented in over 50% of *Campylobacter* isolates in Qatar^32^ and other Gulf countries.^33^,^34^ Furthermore, the effect of antibiotic treatment in prolonging the carrier state for nontyphoidal *Salmonella* may be particularly detrimental among our study population given the risk of person-to-person transmission in close-contact settings.

Our study has certain strengths and limitations. The prospective approach of our study allowed us to ensure that all patients clinically diagnosed with infectious diarrhea were offered the opportunity to enroll in the study and that formed stools were excluded from PCR testing. Retrospective studies are limited by these elements, as the likelihood of stool testing is known to vary among physicians regardless of patient characteristics and laboratory protocols for stool testing may not always be strictly enforced.^2^ Various factors may have influenced the frequencies of the microbes detected in our study, including our chosen case definition, seasonal variation in the incidence of certain pathogens,^32^ and the unique study setting and population. Transportation to the study clinic may have also been a barrier for some migrant workers, which may have biased the pathogen distribution against those that cause more mild illness.^20^ Because of an administrative issue, enrollment into our study was suspended for 19 weeks, which precluded us from reaching our planned sample size. Consequently, the precision of our estimates of the prevalence of the various pathogens was less than anticipated and we were likely underpowered to detect associations with putative risk factors. For logistical reasons, our study did not include a control group, which would have enabled us to compare the frequency of detection of pathogens, particularly the diarrheagenic *E. coli* pathotypes, in asymptomatic persons and those with diarrhea. We also did not compare the performance of the FilmArray^®^ PCR to traditional stool diagnostic methods such as culture, enzyme immunoassay, or uniplex PCR. Such studies have been published previously, however, and demonstrate that the FilmArray^®^ PCR has high sensitivity and specificity compared with traditional methods.^9^,^22^ Finally, although our survey was modified from the validated Minnesota Questionnaire with the input of key stakeholders at the study clinic and the Qatar Ministry of Public Health, the survey was not validated before being implemented for the study.

In summary, use of multiplex PCR enabled the detection of one or more pathogens in 62.7% of cases of infectious diarrhea among the migrant worker population in Qatar, with *Salmonella* and diarrheagenic *E. coli*, the most commonly identified pathogens. Our study illustrates the utility of this diagnostic platform in epidemiologic studies and serves as a foundation for future research to understand the epidemiology and risk factors for infectious diarrhea among the migrant worker population. Further research is needed to understand the optimal use and interpretation of the FilmArray^®^ PCR in the diagnosis of infectious diarrhea.

## Figures and Tables

**Table 1:** The demographic characteristics of study participants

Characteristic	*n* (%) (*N* = 126)
Median age (IQR) in years	33 (27–39)
Male sex	126 (100)
Country of origin	
Nepal	37 (29.3)
India	36 (28.5)
Bangladesh	20 (15.9)
Sri Lanka	19 (15.1)
Philippines	6 (4.8)
Ethiopia	2 (1.6)
Pakistan	2 (1.6)
Egypt	1 (0.8)
Jordan	1 (0.8)
Kenya	1 (0.8)
Syria	1 (0.8)
Type of work	
Construction related*	106 (84.1)
Other†	20 (15.9)
Shift time	
Day	122 (96.8)
Night	3 (2.4)
Day and night	1 (0.8)
Dwelling	
Worker camp	121 (96.0)
Private apartment	4 (3.2)
Private house	1 (0.8)
Number of roommates	
0	2 (1.6)
1–3	35 (27.8)
4–6	46 (36.5)
7–9	38 (30.2)
10 or more	5 (3.9)
Shared bathroom	122 (96.8)
International travel within 21 days of presentation‡	8 (6.3)

IQR = interquartile range.

* Construction-related work includes carpenter (*N* = 15), cleaner (*N* = 1), construction (*N* = 24), duct installer (*N* = 5), electrician (*N* = 13), fabricator (*N* = 1), foreman (*N* = 3), heavy equipment operator (*N* = 4), helper (*N* = 3), insulation technician (*N* = 2), mason (*N* = 7), metal worker (*N* = 9), painter (*N* = 9), pipefitter (*N* = 3), plumber (*N* = 5), and welder (*N* = 2).

† Other work includes auto electrician (*N* = 1), chemical sprayer (*N* = 1), driver (*N* = 8), mechanic (*N* = 4), nurse (*N* = 3), storekeeper (*N* = 2), and tailor (*N* = 1).

‡ Countries traveled to include India (*N* = 4), Bangladesh (*N* = 2), and Nepal (*N* = 2).

**Table 2:** Clinical characteristics of study participants

Characteristic	*n* (%)*
Self-rated baseline health	
Excellent	76 (60.3)
Very good	27 (21.4)
Good	14 (11.1)
Fair	7 (5.6)
Poor	2 (1.6)
Current medical comorbidity†	9 (7.2)
History of intra-abdominal surgery‡	6 (4.8)
Antibiotic use before diarrheal illness	1 (0.8)
Known contact with another person with diarrheal illness	4 (3.2)
Acid-suppressive or immunosuppressive medication use	0 (0)
Location of treatment	
Clinic	97 (77.0)
Emergency department	29 (23.0)
Median duration of symptoms (IQR) in days	2 (2–3)
Symptoms	
Diarrhea	126 (100)
Abdominal cramps	103 (81.8)
Fatigue	97 (77.0)
Fever	53 (42.1)
Body aches	52 (41.3)
Headache	50 (39.7)
Chills	43 (34.1)
Vomiting	33 (26.2)
Nausea	31 (24.6)
Bloody diarrhea	7 (5.6)
≥ 6 stools per day	69 (54.8)
Temperature ≥ 38.5°C (*N* = 107)	9 (8.4)
Heart rate ≥ 90 beats per minute (*N* = 107)	28 (26.2)
Systolic blood pressure < 90 mmHg (*N* = 121)	1 (0.8)

IQR = interquartile range.

* *N* = 126 unless otherwise indicated.

† Medical comorbidity includes diabetes (*N* = 7), hypertension (*N* = 2), and hyperlipidemia (*N* = 1).

‡ All had undergone appendectomy > 2 years before presentation.

**Table 3:** Laboratory and microbiology test results

Characteristic	*n* (%)
Blood count (*N* = 31)	
White blood cells ≥ 15,000/mm^3^	7 (22.6)
Hematocrit < 40%	2 (6.5)
Platelet < 150,000/μL	1 (3.2)
Stool microscopic exam (*N* = 125)*	
≥ 1 fecal leukocytes/high-power field	113 (90.4)
> 5 leukocytes/high-power field	52 (41.6)
> 25 leukocytes/high-power field	26 (20.8)
Red blood cells present	52 (41.6)
Mucous present	49 (39.2)
*Entamoeba* sp.	17 (13.6)
*Giardia lamblia*	9 (7.2)
*Strongyloides stercoralis*	2 (1.6)
*Enterobius vermicularis*	1 (0.8)
*Ascaris lumbricoides*	1 (0.8)
Hookworm	1 (0.8)
Stool culture positive for *Salmonella/Shigella* (*N* = 5)	0
FilmArray^®^ Gastrointestinal PCR results† (*N* = 126)	
Positive for any pathogen	79 (62.7)
Pathogen prevalence (*N* = 126)	
Enteropathogenic *Escherichia coli*	31 (24.6)
*Salmonella*	28 (22.2)
Enteroaggregative *E. coli*	19 (15.1)
*Giardia lamblia*	12 (9.5)
Enterotoxigenic *E. coli* lt/st	11 (8.7)
*Shigella/*enteroinvasive *E. coli*	11 (8.7)
*Campylobacter*	9 (7.1)
Shiga-like toxin-producing *E. coli* stx1/stx2	8 (6.4)
*E.coli* 0157	1 (0.8)
Norovirus GI/GII	5 (4.0)
Rotavirus A	3 (2.4)
*Plesiomonas shigelloides*	2 (1.6)
Astrovirus	1 (0.8)
Adenovirus F 40/41	1 (0.8)
*Cyclospora cayetanensis*	1 (0.8)
Co-detection of ≥ 2 pathogens per positive sample (*N* = 79)	39 (49.3)
2 pathogens	25 (64.1)
3 pathogens	8 (20.5)
4 pathogens	4 (10.2)
5 pathogens	1 (2.6)
6 pathogens	0 (0)
7 pathogens	1 (2.6)
Received antibiotic for treatment of diarrhea‡	85 (67.5)
Metronidazole	62 (49.2)
Ciprofloxacin	45 (35.7)
Trimethoprim-sulfamethoxazole	8 (6.4)
Received IV fluids (*N* = 126)	28 (22.2)

IV = intravenous; PCR = polymerase chain reaction.

* Denotes maximum number of leukocytes or red blood cells seen on any high-power field.

† No detections of sapovirus, *Entamoeba histolytica*, Cryptosporidium, *Clostridium difficile*, *Vibrio* spp., or *Yersinia enterocolitica.*

‡ This refers to the number of subjects out of the total study cohort (*N* = 126) who received an antibiotic by their physician for treatment of diarrhea.

**Table 4:** Univariate analysis of the prevalence of various demographic and clinical characteristics in patients with positive and negative multiplex PCR results*

Characteristic	Positive PCR	Negative PCR	OR (95% CI)	*P* value
	*n* (%) (*N* = 79)	*n* (%) (*N* = 47)		
Demographic				
Country				
Nepal	21 (26.6)	16 (34.0)	0.7 (0.3–1.7)	0.37
India	27 (34.2)	9 (19.1)	2.1 (0.9–5.9)	0.07
Bangladesh	12 (15.2)	8 (17.0)	0.9 (0.3–2.7)	0.79
Sri Lanka	11 (13.9)	8 (17.0)	0.8 (0.3–2.5)	0.64
Philippines	4 (5.1)	2 (4.3)	1.2 (0.2–13.7)	0.84
International travel within 21 days of presentation	6 (7.6)	2 (4.3)	1.8 (0.3–19.4)	0.46
Living with ≥ 6 roommates	33 (42.9)	17 (36.2)	1.3 (0.6–3.0)	0.46
Meals prepared at home†	54 (68.3)	35 (74.5)	0.7 (0.3–1.8)	0.47
Cooking stove in home	56 (70.1)	31 (67.4)	1.1 (0.5–2.8)	0.68
Refrigerator in home	43 (54.4)	24 (52.2)	1.1 (0.5–2.4)	0.81
Clinical				
Location of treatment				
Emergency department	21 (26.9)	8 (17.0)	1.8 (0.7–5.2)	0.20
Symptom duration > 2 days	19 (24.1)	13 (27.7)	0.8 (0.3–2.1)	0.65
Symptoms				
Abdominal cramps	70 (88.6)	33 (70.2)	3.3 (1.2–9.5)	0.01
Fatigue	62 (78.5)	35 (74.5)	1.3 (0.5–3.1)	0.60
Fever	36 (45.6)	17 (36.2)	1.5 (0.7–3.3)	0.30
Chills	29 (36.7)	14 (30.0)	1.4 (0.6–3.2)	0.43
Headache	36 (45.6)	14 (29.8)	1.9 (0.9–4.6)	0.08
Body aches	37 (46.8)	15 (31.9)	1.9 (0.8–4.3)	0.10
Nausea	24 (30.3)	7 (14.9)	2.5 (0.9–7.5)	0.05
Vomiting	24 (30.3)	9 (19.2)	1.8 (0.7–5.0)	0.16
Bloody diarrhea	5 (6.3)	2 (4.2)	1.5 (0.2–16.5)	0.62
≥ 6 stools per day	45 (56.9)	24 (51.1)	1.3 (0.6–2.8)	0.52
Temperature ≥ 38.5°C (*N* = 107)	7 (10.1)	2 (5.2)	2.0 (0.4–21.0)	0.38
Heart rate ≥ 90 beats per minute (*N* = 107)	23 (33.3)	5 (13.2)	3.3 (1.1–12.2)	0.02
Laboratory data (*N* = 125)				
≥ 1 fecal leukocytes/high-power field	70 (89.7)	43 (91.5)	0.8 (0.2–3.3)	0.75
> 5 leukocytes/high-power field	33 (42.3)	19 (40.4)	1.1 (0.5–2.4)	0.84
> 25 leukocytes/high-power field	15 (19.2)	11 (23.4)	0.8 (0.3–2.1)	0.58
Red blood cells present	33 (42.3)	19 (40.4)	1.1 (0.5–2.4)	0.84
Mucous present	34 (43.6)	15 (31.9)	1.6 (0.7–3.8)	0.20
Treatment				
Receipt of any empiric antimicrobial	56 (70.9)	29 (61.7)	1.5 (0.6–3.5)	0.29

CI = confidence interval; OR = odds ratio; PCR = polymerase chain reaction.

* The reference group for each characteristic was all study subjects who did not report that characteristic. The reference group for Nepal, for example, included subjects from all other countries.

† Compared with meals prepared at workplace cafeteria, catering company, or restaurant.
